# Hematoma Mimicking Breast Cancer on CT Scan and Breast Ultrasound

**DOI:** 10.7759/cureus.9099

**Published:** 2020-07-09

**Authors:** Quan D Nguyen, Andrea Tenreiro, James T Roberts, Anahita Tavana, Angelica S Robinson

**Affiliations:** 1 Radiology, University of Texas Medical Branch, Galveston, USA; 2 Diagnostic Radiology, University of Texas Medical Branch, Galveston, USA

**Keywords:** breast cancer, hematoma, ct scan, breast mass, ultrasound, mammogram, benign

## Abstract

There are many benign breast lesions that mimic breast cancer on breast imaging. Postlumpectomy scar, hematoma, fat necrosis, diabetic mastopathy, and granulomatous mastitis are examples of benign breast lesions that have suspicious breast imaging findings. Mammogram and breast ultrasound are the imaging studies to evaluate breast findings. CT scan is not used to evaluate breast findings because it delivers high radiation dose to the breast, and breast tissue is often confused as breast masses on CT scan. The following case demonstrates an incidentally detected breast mass on CT scan performed to assess for pulmonary embolism. The CT scan and subsequent breast ultrasound both demonstrated suspicious breast imaging findings. Final pathology from ultrasound-guided biopsy revealed hematoma. This benign finding was concordant with the patient’s medical history of cirrhosis with low platelet count and medication history of warfarin.

## Introduction

CT is frequently the first radiologic study to detect an abnormal breast lesion due is increasing use. It generally reveals insufficient detail for diagnosis of distinct pathology in the breast. However, there are certain imaging characteristics on CT that may provide diagnostic clues regarding the possibility of an incidental lesion to be suspicious [1]. Suspicious features include irregular margins, shape, and rim enhancement [2]. No definite characteristics have been found on CT to be accurate predictors of benignity [1]. Mammography and stability over time will ultimately be required to reliably characterize a lesion, with or without malignant features, incidentally detected on CT. Postsurgical changes in the breast can frequently mimic malignancy, granting importance to knowing the patients surgical and medical history when assessing incidental breast lesions.

## Case presentation

**Figure 1:**
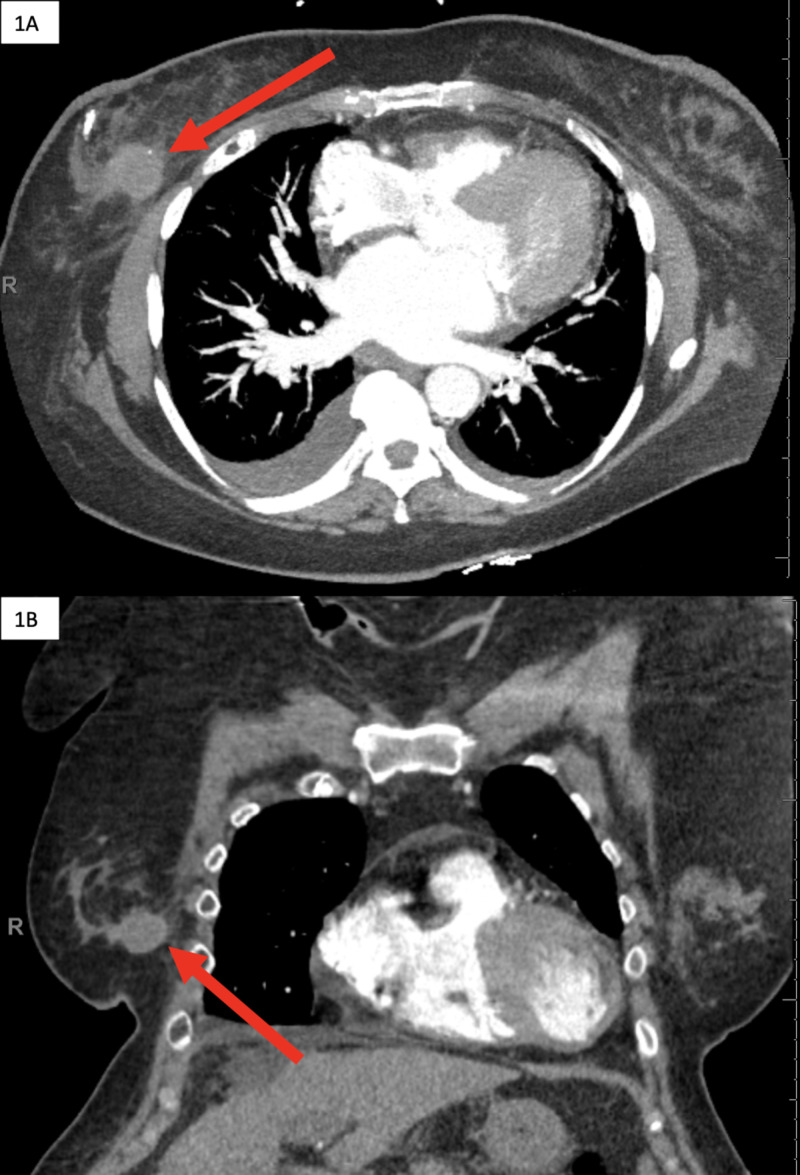
Pulmonary angiogram chest CT performed in the emergency department Axial (A) and coronal (B) images demonstrate incidental finding of isoattenuating mass with subtle rim enhancement (red arrows) in the right breast.

A 69-year-old female presented to the emergency department with shortness of breath. Past medical history was significant for grade 3 invasive ductal carcinoma (IDC) of the right breast with associated ductal carcinoma in situ (DCIS) diagnosed six years before presentation, status postlumpectomy and chemoradiation, diabetes mellitus type 2, hypertension, and cirrhosis secondary to nonalcoholic steatohepatitis. The patient’s multiple medications include aspirin and warfarin. Workup upon arrival included a chest x-ray and CT chest pulmonary angiogram. Pertinent findings included a circumscribed, isoattenuating mass in the right breast measuring 2.4 x 2.5 cm with subtle rim enhancement (anteroposterior by transverse dimension) (Figure 1).

**Figure 2:**
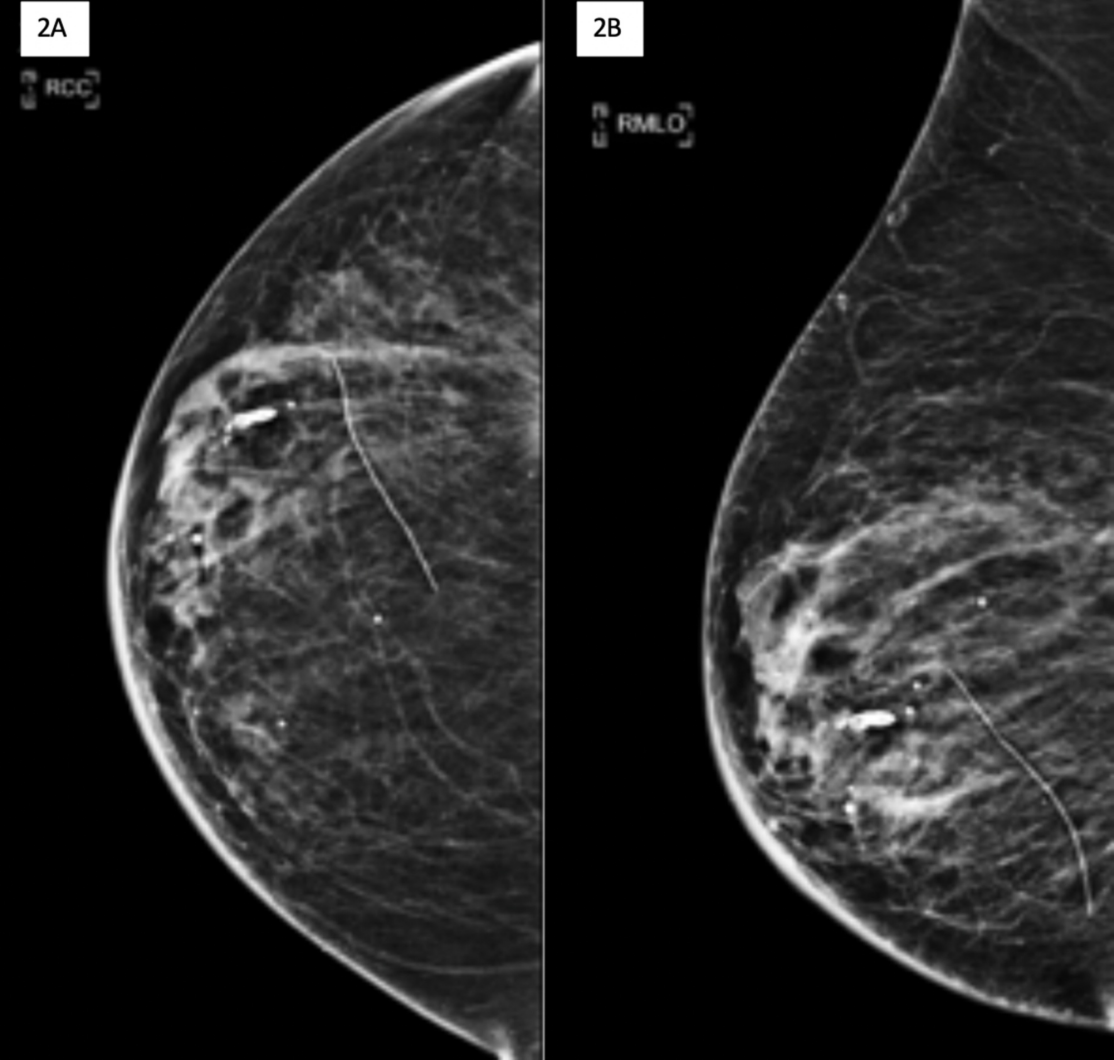
Most recent screening mammogram two weeks prior to the abnormal CT scan Right craniocaudal (A) and mediolateral oblique (B) views from most recent mammogram. There are postlumpectomy changes with no suspicious masses, calcifications, or abnormal findings. The abnormal CT finding was far posterior and thus not included in the field of view on mammogram.

Most recent screening mammogram two weeks prior to the abnormal CT scan was Breast Imaging Reporting and Data System (BI-RADS) category 2 considered as benign for postlumpectomy changes (Figure 2). Of note, the abnormal CT finding was far posterior and thus not included in the field of view on mammogram.

**Figure 3:**
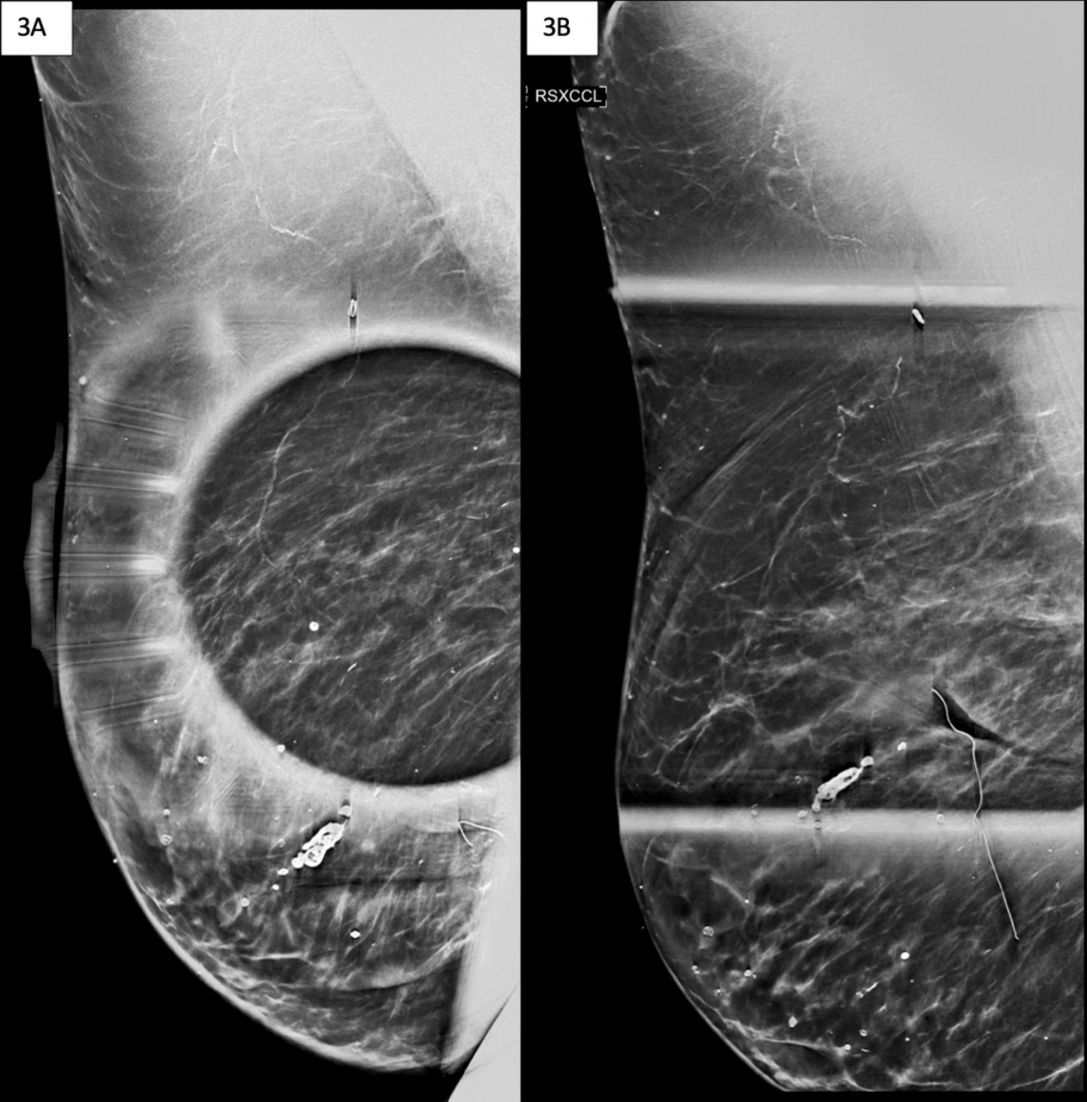
Images of diagnostic mammogram Image A details right breast spot compression in the medial lateral oblique view (RSMLO) of the lumpectomy bed. Image B details right breast spot compression exaggerated in the craniocaudal view (RSXCCL) of the lumpectomy bed. There are postlumpectomy changes with no suspicious masses, calcifications, or abnormal findings. Again, the abnormal CT finding was far posterior and thus not included in the field of view on mammogram.

Diagnostic mammogram of the right breast to further evaluate the suspicious mass seen on CT scan was performed. Findings include postlumpectomy changes in a heterogeneously dense breast (Figure 3). Again, the abnormal CT finding was far posterior and thus not included in the field of view on mammogram.

**Figure 4:**
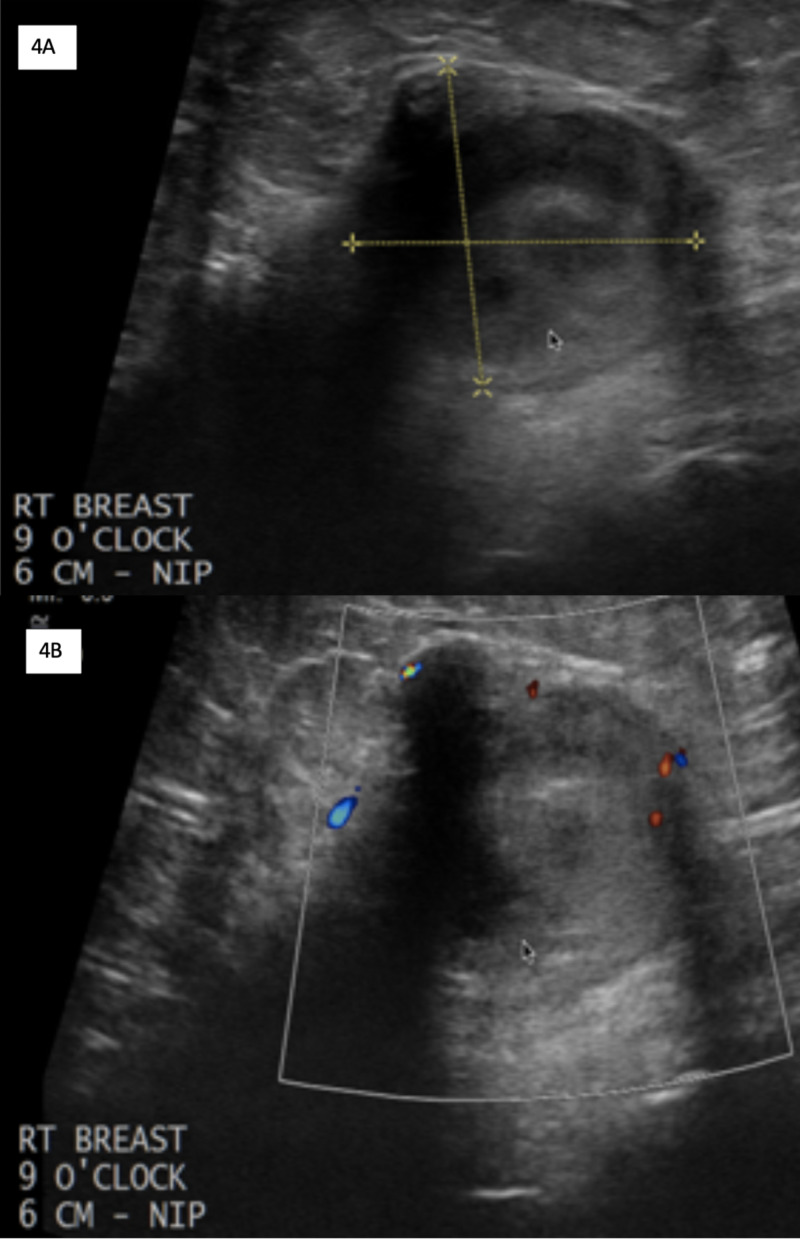
Ultrasound of the right breast Images demonstrate a circumscribed, heterogeneous mass (measured by yellow lines, A) at 9 o’clock, 6 cm from nipple, measuring 21 x 20 x 25 mm (A) with minimal color. Image B displays Doppler flow.

Ultrasound images of the right breast mass were obtained (Figure 4). A nonparallel, heterogeneous mass with circumscribed margins was seen at 9 o'clock, 6 cm from the nipple. This correlated with the recent CT abnormal finding. Minimal associated vascularity was noted. No lymphadenopathy was present in the axillary, infraclavicular, supraclavicular, or internal mammary chain region (not pictured). 

Core needle biopsy of the mass was performed. Pathology revealed an organizing hematoma with hemosiderin-laden macrophages, multinucleated giant cell reaction, and dense stromal fibrosis. Immunostains performed demonstrated CD31, CD34, and p63 positivity, D2-40 negativity, and Ki-67 with scattered cell stains which supported pathology diagnosis.

## Discussion

Approximately 8%-10% of women with breast cancer will develop recurrence [3-5]. Therefore, when assessing a new breast mass in patient with history of malignancy, dedicated imaging is required. Breast hematomas can develop after surgery, biopsy, or trauma [1,6]. Therefore, clinical history is important in the workup of these lesions. Breast hematomas have a variety of appearances on CT: may be obscured by edema in the immediate postsurgical/postbiopsy period, a well-circumscribed high-density fluid attenuation mass, an ill-defined mass, or a mass with spiculated margins due to fibrotic posthealing changes which typically regress over time [1,7].

The emergency room radiologist described the right breast mass as a 2-cm mass very concerning for malignancy on the CT scan report. The breast imaging radiologist on subsequent breast ultrasound described the right breast mass as a 25-mm nonparallel, heterogeneous mass with circumscribed margins seen at 9 o'clock, 6 cm from the nipple; given the patient's history of triple negative breast cancer (diagnosed at an outside facility approximately six years ago), this was highly concerning for malignancy (BI-RADS category 5). In short, the right breast mass was suspicious on both CT scan and breast ultrasound.

BI-RADS category 5 is assigned to breast imaging findings that are highly suggestive of malignancy with at least 95% likelihood of cancer [8]. Typically, when BI-RADS category 5 is assigned, the patient will be referred for surgical excision regardless of the pathology result. Although this case was assessed as BI-RADS category 5 by the breast imaging radiologist, the patient was not referred for surgical excision when the pathologist reported hematoma. Hematoma can mimic breast cancer on CT scan and breast ultrasound. Furthermore, the patient’s medical history of cirrhosis with low platelet counts and medication history of warfarin explains the hematoma as the cause for the breast mass seen on breast imaging.

## Conclusions

When pathology for a highly suspicious finding comes back as benign, the breast imaging radiologist must determine whether the results are concordant returning the patient to annual screening mammogram or discordant sending the patient for further surgical excision. There are many benign entities that mimic breast cancer on breast imaging studies, including postlumpectomy scar, hematoma, fat necrosis, diabetic mastopathy, and granulomatous mastitis. Therefore, clinical history must be considered when coming up with a differential diagnosis and when reviewing pathology for concordance.
